# GWEP Successes and Lessons Learned From Making Communities Age Friendly

**DOI:** 10.1093/geroni/igab046.1905

**Published:** 2021-12-17

**Authors:** Katherine Thompson, Angela Catic

## Abstract

Geriatrics Workforce Enhancement Programs (GWEPs), funded by the Health Resources and Services Administration have a strong focus on age friendly care and community engagement. With a wide range of populations, locales, and health systems served, GWEPs have significant experience working with a wide variety of communities to implement age friendly care. In this symposium, we present successes and lessons learned from GWEP projects representing diverse populations and approaches to achieving age friendly communities. For instance, one GWEP is utilizing Patient Priorities Care to lay the framework for What Matters in clinical decision-making. Another GWEP is focusing on What Matters by uniquely embedding Area Agencies on Aging care coordinators within primary care settings to invite the participation of aging patients in advance care planning, among other health interventions. A third GWEP is using the 4Ms to educate patients and caregivers in geriatric psychiatry clinics in a population of veterans. Another GWEP is pairing Age Friendly Health System efforts within a health system with community-based efforts to become an age friendly and dementia friendly city. A final GWEP is using multiple educational modalities to create Age-Friendly Communities and assure that health systems, community-based organizations, and older adults and families are educated about the 4Ms. By exploring successes and lessons learned in making communities age friendly, we can improve existing and future programs centered on age friendly care for older adults.

